# Genome-wide identification of specific oligonucleotides using artificial neural network and computational genomic analysis

**DOI:** 10.1186/1471-2105-8-164

**Published:** 2007-05-22

**Authors:** Chun-Chi Liu, Chin-Chung Lin, Ker-Chau Li, Wen-Shyen E Chen, Jiun-Ching Chen, Ming-Te Yang, Pan-Chyr Yang, Pei-Chun Chang, Jeremy JW Chen

**Affiliations:** 1 Department of Computer Science, National Chung-Hsing University, Taichung, Taiwan, ROC; 2 Institute of Biomedical Sciences, National Chung-Hsing University, Taichung, Taiwan, ROC; 3 Institute of Statistical Science, Academia Sinica, Taipei, Taiwan, ROC; 4 Institute of Molecular Biology, National Chung-Hsing University, Taichung, Taiwan, ROC; 5 NTU Center for Genomic Medicine, National Taiwan University College of Medicine, Taipei, Taiwan, ROC; 6 Departments of Biotechnology and Bioinformatics, Asia University, Taichung, Taiwan, ROC

## Abstract

**Background:**

Genome-wide identification of specific oligonucleotides (oligos) is a computationally-intensive task and is a requirement for designing microarray probes, primers, and siRNAs. An artificial neural network (ANN) is a machine learning technique that can effectively process complex and high noise data. Here, ANNs are applied to process the unique subsequence distribution for prediction of specific oligos.

**Results:**

We present a novel and efficient algorithm, named the integration of ANN and BLAST (IAB) algorithm, to identify specific oligos. We establish the unique marker database for human and rat gene index databases using the hash table algorithm. We then create the input vectors, via the unique marker database, to train and test the ANN. The trained ANN predicted the specific oligos with high efficiency, and these oligos were subsequently verified by BLAST. To improve the prediction performance, the ANN over-fitting issue was avoided by early stopping with the best observed error and a k-fold validation was also applied. The performance of the IAB algorithm was about 5.2, 7.1, and 6.7 times faster than the BLAST search without ANN for experimental results of 70-mer, 50-mer, and 25-mer specific oligos, respectively. In addition, the results of polymerase chain reactions showed that the primers predicted by the IAB algorithm could specifically amplify the corresponding genes. The IAB algorithm has been integrated into a previously published comprehensive web server to support microarray analysis and genome-wide iterative enrichment analysis, through which users can identify a group of desired genes and then discover the specific oligos of these genes.

**Conclusion:**

The IAB algorithm has been developed to construct SpecificDB, a web server that provides a specific and valid oligo database of the probe, siRNA, and primer design for the human genome. We also demonstrate the ability of the IAB algorithm to predict specific oligos through polymerase chain reaction experiments. SpecificDB provides comprehensive information and a user-friendly interface.

## Background

DNA microarray is a powerful tool in functional genome studies [1-4]. However, it usually generates false positive data as a result of cross-hybridization between highly similar sequences [5-7]. The design approach of polymerase chain reaction (PCR) primer with minimal cross homology is an important technology [8]. In addition, the recent application of siRNAs to silence genes is dependent on the sequence specificity, and the siRNA sequence must be selected carefully to avoid similarity to an unrelated mRNA [9]. Thus, the important issue is finding a way to effectively identify specific oligonucleotides (oligos).

The early design of specific oligos was based mainly on the use of a frequency matrix [10,11]. Subsequently, several approaches were developed to design unique oligos, such as an information-theoretical method based on maximum entropy, which has also been applied to the design of probe sets [12]; a method based on matching the frequency of sequence landscapes, which was used to select optimal oligos for *E. coli*, *S. cerevisiae*, and *C. elegans *[13]; suffix trees, which has been used to select the organism-specific signature oligos [14]; the design of genome-wide specific oligos based on basic local alignment search tool (BLAST) [15]; and the smart filtering technique, which was employed to avoid redundant computation while maintaining accuracy [16].

However, these processes still take a long time to identify specific oligos. It is quite obvious that the high-throughput prediction of specific oligos is important for application in large-scale gene analysis. Recently, a method for unique oligo discovery that was a modification of a central pattern partitioning principle was published [17]. This method analyzed 17 complete genomes representing a wide range of both prokaryotic and eukaryotic organisms. However, huge genomes, such as the human genome, were not processed in this report.

An artificial neural network (ANN) is a popular learning approach that effectively handles noise and complex relationships in a robust way [18]. In previous studies, ANNs were employed to process a broad range of input parameters on sequence information, such as base composition and binding properties, to predict anti-sense oligos targeting the mRNA [19,20]. In addition, ANNs have been widely applied to various research fields in biology such as clinical cancer research [21], protein function prediction [22], protein classification [23], and cancer classification [24].

In this study, we present a novel and efficient algorithm that integrates ANN and BLAST, named the IAB algorithm, to identify specific oligos from the Institute for Genomic Research (TIGR) human gene index (HGI) and rat gene index (RGI) databases. Furthermore, we applied the IAB algorithm to construct SpecificDB, a web server that provides users with the appropriate hybridization probe, siRNA, and primer for the HGI sequences. These tools will be of great benefit to functional genomics studies.

## Results

### Construction of unique marker database and the architecture of ANN

**Figure 1:**
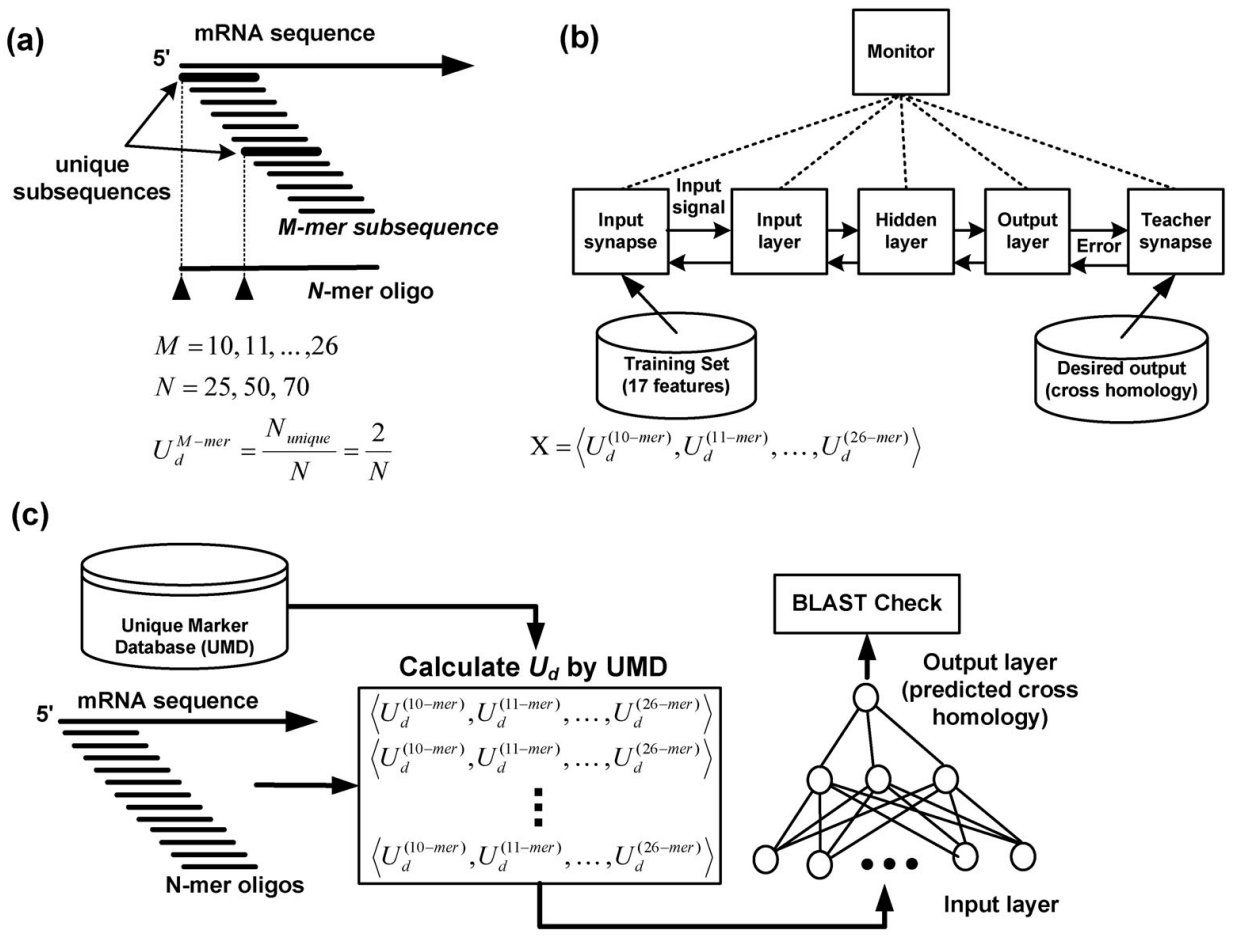
**Integration of ANN and BLAST (IAB)**. (**a**) Calculation of the density of unique subsequence: the solid triangles mark the starting position of the unique subsequences in an N-mer oligo, and the count of the solid triangles is the number of unique subsequences. Thus, the M-mer *U*_*d *_can be calculated from the number of unique subsequences. (**b**) ANN training: there were 17 input nodes in the ANN for the input vector (10-mer ~ 26-mer *U*_*d*_) that is calculated in (a). In addition, the cross homology identified by WU-BLAST was as the desired output. The monitor object represents the central point that contains all of the parameters needed for other components to work properly. (**c**) IAB algorithm architecture: for each sliding N-mer oligo, the input vector (10-mer ~ 26-mer *U*_*d*_) calculated by the unique maker database (UMD) was delivered to the ANN for cross homology prediction. The selected oligos were checked by BLAST after filtering by ANN scores.

**Figure 2:**
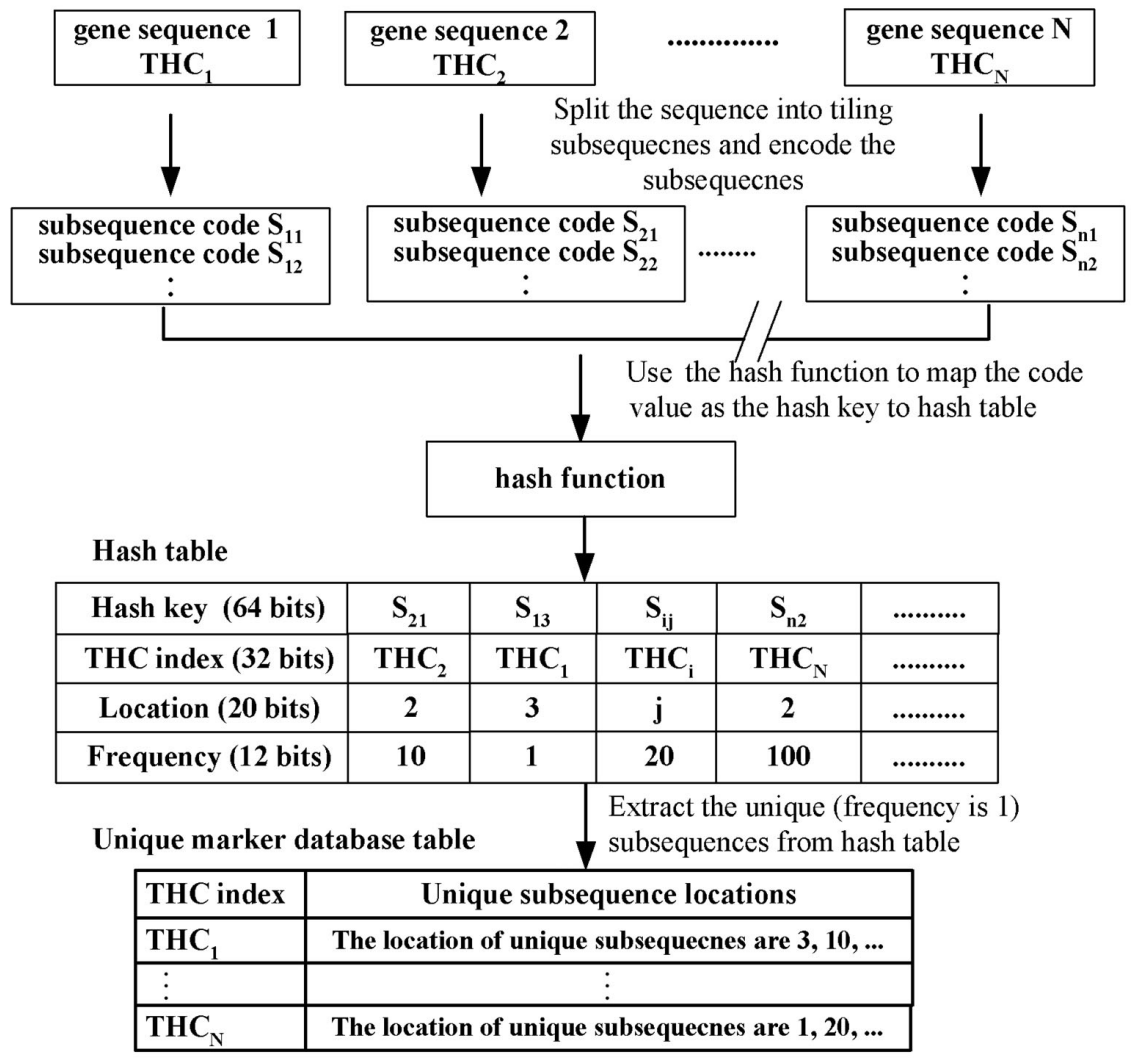
**A flowchart depicting the procedures for the creation of a UMD**. The subsequences and their complementary sequences for all of the genes in the database were encoded and placed in the hash table using the coding number of the subsequences as the hash key. If the subsequence appears only once, the subsequence is treated as unique, and then its location will be stored in the marker tables of UMD.

The input vector of the ANN was derived from the density of the unique subsequences (*U*_*d*_) between 10-mer and 26-mer (Figure 1). In our previous study, an algorithm with 15-mer *U*_*d *_was developed to speed up the identification of a specific probe [1] and we extended and enhanced the algorithm in this study. We established a unique marker database (UMD) to store the positions of all unique subsequences for the entire TIGR HGI tentative human consensus (THC) database and RGI tentative consensus (TC) database [25]. The UMD included of 10-mer ~ 26-mer unique marker subsequences and its workflow is illustrated in Figure 2. Determining the appropriate weights of 10-mer ~ 26-mer *U*_*d *_for the specific oligo prediction was a difficult issue, and the optimal weights depended on the sequence constitution of the genome.

In this study, to determine the optimal weights of 10-mer ~ 26-mer *U*_*d*_, ANN was utilized in our algorithm to predict the oligo specificity. Table 1 shows the number of unique markers and the average density of 10-mer ~ 26-mer unique subsequences in the UMD of HGI and RGI. The results reveal that if the length of the screening subsequence (N-mer) was less than 12-mer, most subsequences were not unique in a large database (*U*_*d *_approximates to 0). On the other hand, if the N-mer was more than 24-mer, many subsequences would be unique (*U*_*d *_of HGI approximates to 0.23 and *U*_*d *_of RGI approximates to 0.49). Therefore, the construction of the unique marker subsequences with 10-mer ~ 26-mer in the UMD was reasonable. The architecture of ANN's backward propagation is shown in Figure 1b. There were 17 input nodes in the ANN for the *U*_*d *_of 10-mer ~ 26-mer. The cross homology calculated by WU-BLAST [26] for each input sequence was as the desired output.

**Table 1:** The number of unique markers and the average density of 10-mer ~ 26-mer subsequences in the database of HGI and RGI.

*N-mer*	*HGI*^*a*^		*RGI*^*b*^	
	Unique Markers	Average *U*_*d*_^c^	Unique Markers	Average *U*_*d*_
10-mer	0	0.000000	4	0.000000
11-mer	6	0.000000	9,669	0.000120
12-mer	24,164	0.000054	457,681	0.005675
13-mer	1,124,491	0.002513	4,167,009	0.051672
14-mer	9,488,257	0.021202	15,364,798	0.190527
15-mer	35,768,666	0.079925	27,962,463	0.346742
16-mer	66,958,259	0.149618	34,903,375	0.432811
17-mer	85,694,125	0.191484	37,636,118	0.466698
18-mer	94,052,393	0.210160	38,645,904	0.479219
19-mer	97,689,916	0.218288	39,061,604	0.484374
20-mer	99,559,386	0.222466	39,276,816	0.487043
21-mer	100,781,233	0.225196	39,417,681	0.488789
22-mer	101,743,851	0.227347	39,522,956	0.490095
23-mer	102,583,079	0.229222	39,608,919	0.491161
24-mer	103,348,039	0.230931	39,682,141	0.492069
25-mer	104,057,929	0.232518	39,744,826	0.492846
26-mer	104,724,128	0.234006	39,800,164	0.493532

^a^HGI, human gene index; ^b^RGI, rat gene index; ^c^*U*_*d*_, the density of the unique subsequences.

### Over-fitting and validation

**Figure 3:**
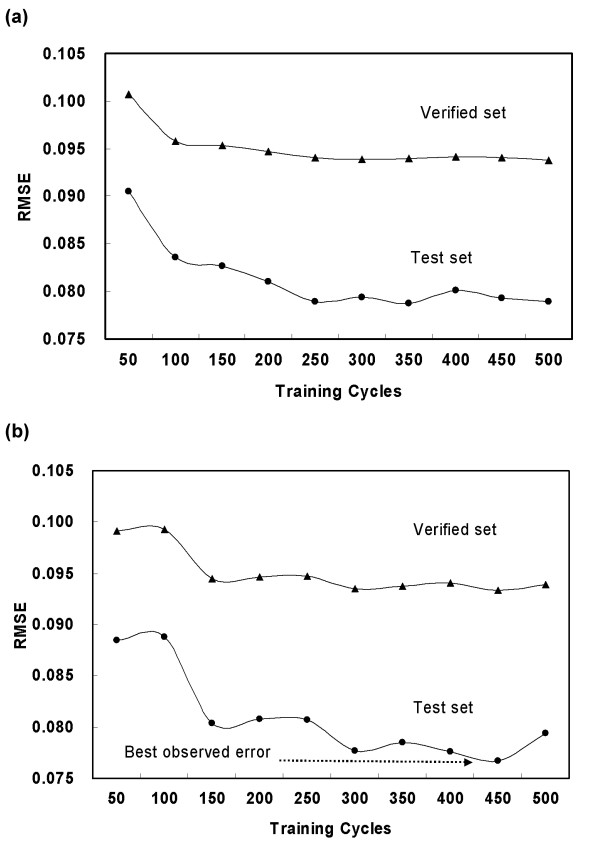
**The performance profiles with different training epochs for test and validation sets**. The ANNs were trained with different numbers of hidden layer nodes as follows: (**a**) 16 hidden nodes, and (**b**) 22 hidden nodes. The profiles show that the optimal performance for the test and validation sets did not occur at the maximum training epochs. RMSE became more stable and relatively lower when the training epoch number was between 350 and 500. The best observed error for the test set is RMSE 0.0767 at 450 (b).

If too much training is applied to the training set, over-fitting of the ANN will occur, which means that it will be fitted precisely to the training set and thereby lose accuracy in the independent test set. Over-fitting would be expected with sufficiently large ANNs and sufficiently "successful" training [27]. The results of over-fitting tests in this study revealed that the over-fitting effect was dependent on the number of hidden layer nodes (Figure 3).

**Figure 4:**
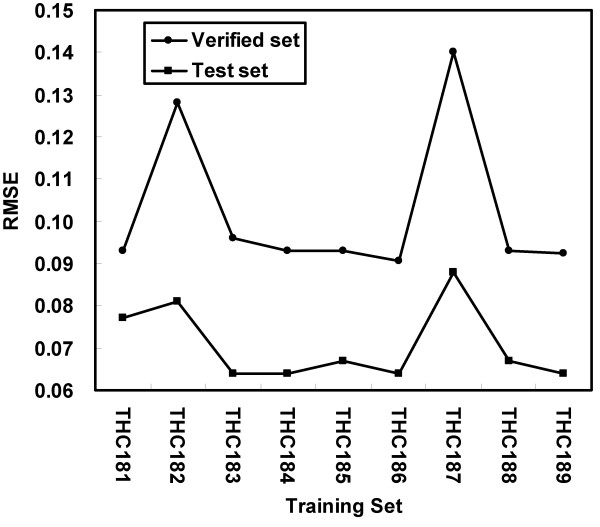
**The performance profiles of a k-fold validation**. The RMSEs of the test and validation sets have the same trend, which signals the generality of the result. The best performance occurred in training set TS_THC186 _for both the test and validation sets.

Our results show that the over-fitting effect of the ANN performance with 22 hidden nodes (Figure 3b) is more pronounced than that with 16 hidden nodes (Figure 3a). Furthermore, root mean square error (RMSE) [28] became quite stable and relatively lower when the training cycle number was between 350 and 500. The best observed error for the test set happened when the training cycle was 450 (RMSE = 0.0767, Figure 3b). Thus, the setting of 450 cycles was applied to all future procedures. Furthermore, k-fold validation was performed on nine training sets [29]. Thus, nine trained ANNs were produced and tested using the independent test set. In addition, we also evaluated the ANN's performance using an independent large-scale validation set. The results show that the RMSEs of the test and validation sets had similar profiles and the best RMSE occurred in the same training set (TS_THC186_) for both the test and validation sets (Figure 4). The consistent profile of both the test and validation sets indicates the stability of the ANN's performance. Thus, the ANN trained by TS_THC186 _was selected for genome-wide identification of the specific oligos.

### Performance comparison for IAB algorithm

To investigate the performance at the various lengths of oligos, 100 THC sequences on which to perform the IAB algorithm, the pure BLAST search, and the BLAST search with *U*_*d *_were randomly selected from the HGI database. Three performance criteria were computed and evaluated, including success rate, average cross homology and execution time. The sensitivity factor is the maximum percentage of sliding oligos that should be screened by BLAST. Thus, the sensitivity factor is used to adjust the sensitivity of the IAB algorithm in this report. Moreover, to investigate the effect of the sensitivity factor on performance, various sensitivity factors were evaluated. The results reveal that the IAB algorithm relative to other approaches had better or equal quality with a sensitivity factor of 0.3 for 70-mer, 50-mer, and 25-mer in the success rate, average cross homology, and execution time (see Table 2). Thus, the sensitivity factor was set at 0.3 in all performance comparisons.

**Table 2:** Performance comparison with and without an artificial neural network.

Oligo length	Procedure	Sensitivity factor	Success rate (%)^a^	Cross homology ^b^	Execution time (hours)
70-mer	IAB ^c^	0.2	94	0.56	0.50
		0.3	95	0.56	0.71
		0.4	95	0.56	0.90
	BLAST with *U*_*d*_^d^		95	0.56	2.13
	Pure BLAST		95	0.61	3.69
50-mer	IAB	0.2	91	0.64	0.20
		0.3	93	0.64	0.31
		0.4	93	0.64	0.32
	BLAST with *U*_*d*_		93	0.64	0.70
	Pure BLAST		93	0.69	2.19
25-mer W = 11^e^	IAB	0.2	94	0.79	0.19
		0.3	94	0.79	0.26
		0.4	94	0.79	0.33
	BLAST with *U*_*d*_		94	0.79	0.78
	Pure BLAST		94	0.80	1.62
25-mer W = 8	IAB	0.2	93	0.80	0.36
		0.3	93	0.80	0.50
		0.4	93	0.80	0.64
	BLAST with *U*_*d*_		93	0.80	1.52
	Pure BLAST		93	0.81	3.51
25-mer W = 5	IAB	0.2	92	0.81	1.00
		0.3	92	0.81	1.39
		0.4	92	0.81	1.79
	BLAST with *U*_*d*_		92	0.81	4.25
	Pure BLAST		92	0.81	9.69

^a^The success rate is the percentage of tentative human consensus sequences on which the procedure can find the specific oligo (where the cross homology is less than the threshold). ^b^The cross homology of a specific oligo is determined by the similarity between the specific oligo and its best homology in the non-target sequence. ^c^IAB, integration of artificial neural network (ANN) and basic local alignment search tool (BLAST).^d^BLAST with *U*_*d*_, BLAST search with the density of unique subsequences. ^e^W is word length in the parameters of BLAST.

In HGI database, the IAB algorithm was executed about 5.2, 7.1, and 6.7 times faster than the pure BLAST search for 70-mer, 50-mer, and 25-mer, respectively (Table 2). In the comparison of the BLAST search with *U*_*d*_, the IAB algorithm performed about 3.0, 2.3, and 3.0 times faster for 70-mer, 50-mer, and 25-mer, respectively. In 25-mer specific oligo design, Table 2 shows that the IAB algorithm decreased execution times by 6.2, 7.0, and 7.0 times for word lengths of 11, 8, and 5, respectively. In RGI database, the IAB algorithm, the pure BLAST search, and the BLAST with *U*_*d *_for 70-mer specific oligos were performed. The IAB algorithm was executed about 7.3 times faster than the pure BLAST search and 2.0 times faster than the BLAST with *U*_*d *_(Table 3). In addition, the BLAST with *U*_*d *_was executed about 3.6 times faster than the pure BLAST search. It is expectable that the BLAST with *U*_*d *_has better performance than the pure BLAST search and the IAB algorithm has better performance than the BLAST with *U*_*d *_algorithm.

**Table 3:** Performance comparison for 70-mer RGI.

Procedure	Sensitivity factor	rate (%)^a^	Cross homology^b^	Execution time (hours)
IAB^c ^for HGI^d^	0.2	99	0.53	0.09
	0.3	99	0.53	0.11
IAB for RGI^e^	0.2	99	0.53	0.10
	0.3	99	0.53	0.12
Pure BLAST		99	0.58	0.87
BLAST with *U*_*d*_^f^		99	0.53	0.24

^*a*^The success rate is the percentage of tentative human consensus sequences on which the procedure can find the specific oligo (where the cross homology is less than the threshold). ^*b*^The cross homology of a specific oligo is determined by the similarity between the specific oligo and its best homology in the non-target sequence. ^*c*^IAB, integration of artificial neural network (ANN) and basic local alignment search tool (BLAST).^d^HGI, human gene index.

^e^RGI, rat gene index. ^*f*^BLAST with *U*_*d*_, BLAST search with the density of unique subsequences.

### Specific oligo web server (SpecificDB)

**Figure 5:**
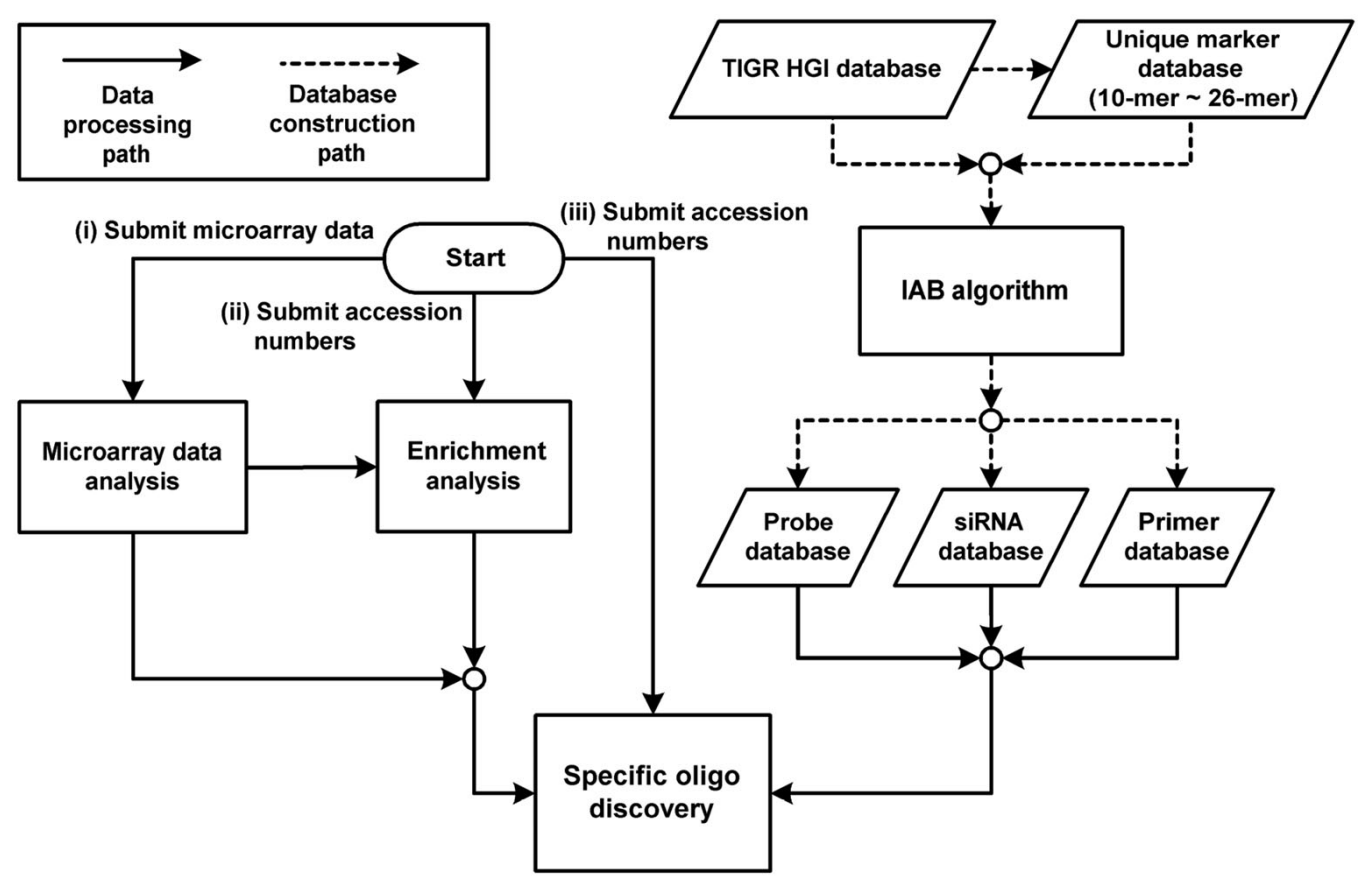
**The architecture of SpecificDB**. The web server SpecificDB includes the probe, siRNA, and primer databases, and integrates the IAB algorithm, microarray data analysis, and enrichment analysis. There are three initial workflows into which users can submit data: (i) microarray data can be submitted to the microarray data analysis component; (ii) GenBank accession numbers or UniGene IDs of a group of genes can be submitted to the enrichment analysis component; and (iii) GenBank accession numbers or UniGene IDs of a group of genes can be submitted to the specific oligo discovery component.

To provide a useful and powerful web server named SpecificDB, the following steps were taken: (1) unique subsequences with 10-mer ~ 26-mer were created in UMD; (2) training, test, and validation sets were prepared; (3) k-fold validation was performed on training sets; (4) the IAB algorithm was implemented; (5) specific oligos including probe, siRNA, and primer were constructed; and (6) the specific oligo database was incorporated into our previous work (a comprehensive web server for the composite regulatory signature database, CRSD) [30] consisting of microarray analysis, motif discovery, and genome-wide iterative enrichment analysis for microRNAs, transcription factors, pathways, and GO annotations. Users can perform microarray data analysis and enrichment analysis to identify a group of interesting genes, and then discover the specific oligos for the probe, siRNA, and primer of these genes in the SpecificDB web server. The architecture of SpecificDB is illustrated in Figure 5, and the server is available at our web site [31].

### Application of IAB algorithm and the demonstration of predicted primers

The IAB algorithm was applied to identify the specific primers of *Xanthomonas campestris pv. Campestris (Xcc) *strain 17 contigs that were constructed and sequenced. To locate all of the genes in *Xcc *strain 17, another similar strain, *Xcc *strain 33913 containing 4,181 genes [32], was employed to perform sequence alignment. The required information and annotation of *Xcc *strain 33913 genome is available in the NCBI database.

We aligned the 4,181 gene sequences against the *Xcc *strain 17 contigs to obtain the annotation data of the contigs. The results showed that the average sequence similarity between the two strains was 94.81%. There were 3,836 genes with similarity in excess of 90% that were selected as the predicted genes in *Xcc *strain 17. In order to avoid the non-specific annealing of predicted primers, three additional genomes (*A. thaliana*, *S. cerevisiae*, and *E. coli*) were merged with the *Xcc *strain 17 contigs to build an integrated nucleotide sequence database that was used for non-specific oligo filtration. A total of 3,569 primer sets were identified from 3,836 genes by using a cross homology threshold of 85% against the integrated nucleotide sequence database [see Additional file 1]. More than 93% of all the genes contained the specific and valid primer sets.

**Figure 6:**
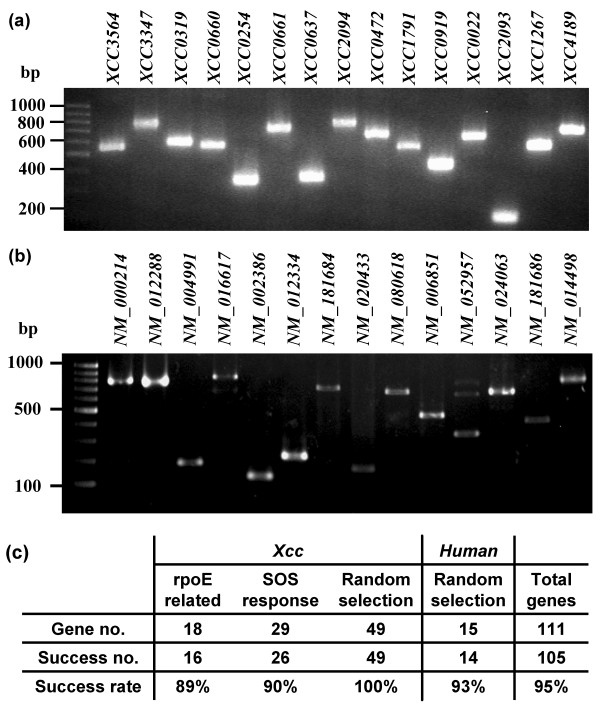
**PCR amplification by using the primers predicted by the IAB algorithm**. 96 *Xcc *and 15 human primer sets were selected for PCR amplification. (**a**) Electrophoretic analysis of *Xcc *PCR products. Representative results of PCR amplification for 15 genes are shown. (**b**) Electrophoretic analysis of human PCR products. Fourteen genes with correct size of PCR products are shown. (**c**) The success rates of PCR amplification for *Xcc *and human are presented. The total success rate was 95%

To demonstrate the applicability of predicted primers, we selected 18 and 29 genes related to SOS response [33] and *rpoE *[34] respectively, as well as 49 randomly selected genes in *Xcc *strain 17 to perform PCR amplification using the primers predicted by the IAB algorithm. In addition, to verify the primer design based on the IAB algorithm for large genomes such as human, we randomly selected 15 human primer sets from our SpecificDB database and performed PCR amplification. A total of 111 PCR reactions (96 for *Xcc *and 15 for human) were performed in twice and followed by electrophoresis analysis. If a PCR product with correct size can be found at least once, we count the result as a success. The PCR results show that the success rate was 95% and 93% for *Xcc *and human, respectively, and all PCR products had correct size. Representative results are shown in Figure 6. In human PCR results, there is one gene (NM_052957) that has multiple bands but a single dominant band with correct size.

## Discussion

Several important techniques in molecular biology, such as siRNA, microarray, and primer design, need short and specific oligos. The prediction of short and specific oligos is essential for these applications [9,35]. The BLAST search can be utilized to deal with the identification of specific oligos [36] but it requires too much computing time to screen all sliding oligos. Thus, a fast and efficient predictor for sequence specificity is needed [37]. In this study, ANN is used as a predictor to filter out the oligos with high cross homology before the BLAST search. Here, a new method for genome-wide identification of specific oligos is developed, and it integrates ANN and BLAST to optimize the sequence analysis by using the densities of the various length unique subsequences.

Early in this study, we investigated repeat frequencies of subsequence (6-mer ~ 12-mer) and Shannon's entropy of subsequence frequencies distributions [38], which are related to the degeneracy of the subsequence coding scheme. However, we did not discover an efficient method to integrate these distributions (unpublished data). In this report, we found that the integration of the 10-mer ~ 26-mer *U*_*d *_and ANN is an efficient approach to predict oligo specificity.

In siRNA design, BLAST is frequently used to determine the specificity of siRNAs. However, BLAST may lose sensitivity and miss important alignment for such short oligos as siRNAs [39,40]. On the other hand, the sensitivity of BLAST depends on the word length parameter so that shorter word lengths may increase sensitivity but decrease execution speed. To investigate the impact of the word length, several word lengths (11, 8, and 5) were employed to evaluate the performance of our algorithm on specific short oligo identification. Table 2 shows that the IAB algorithm enjoys significant improvement in speed for various word lengths. Thus, the shorter word length can be applied to improve sensitivity in the IAB algorithm.

The combination of ANN and *U*_*d *_is a part of the IAB algorithm. The performance comparison of the IAB algorithm and the BLAST search with *U*_*d *_revealed that ANN is an important component in the IAB algorithm (Table 2). The IAB algorithm with appropriate sensitivity factor had lower cross homology and shorter execution time. Although the IAB algorithm only screened a portion of the sliding oligos, it still had better quality than the pure BLAST search that may screen all sliding oligos.

It is difficult to understand the inside workings of an ANN, where learned knowledge is contained in the weight (coefficient) of synapse in the ANN structure. Thus, the ANN is usually treated as a black box [41] and the biological significance inside can not be interpreted. However, ANNs have been applied to various research fields in bioinformatics. In addition, the unique subsequence distribution has also been utilized in various aspects of sequence analysis [1,42]. In this study, we integrated a wide range of unique subsequences (10-mer ~ 26-mer) using the ANN approach to improve the identification of specific oligos. Such a wide range of unique subsequences has not been previously reported.

ANN training for every genome and every length of oligo is inflexible and inconvenient for applications, but our findings indicate that the IAB algorithm may overcome this problem. Table 2 demonstrates the robustness of our algorithm by applying the ANN trained for 70-mer oligos to the prediction of 50-mer and 25-mer specific oligos. Our results show that the predictions for the 50-mer and 25-mer specific oligos have similar performance to that of the 70-mer.

Furthermore, to understand whether the final trained ANN from HGI could be applied to other genomes, we derived the 70-mer training, test, and validation sets from RGI, and performed a k-fold validation method to obtain the best trained ANN. Then, we randomly selected 200 RGI TC sequences to carry out the specific oligo selection by using the trained ANN of HGI and RGI with IAB, as well as the pure BLAST search and the BLAST with *U*_*d*_. The results of the experiments show that trained ANN from both HGI and RGI had almost the same performance in specific oligo selection for the RGI database (Table 3). Therefore, it may be not necessary to perform the complicated procedures of training a new ANN for RGI. Thus, the final trained ANN from HGI has robustness to other genomes such as RGI, and the IAB algorithm can be employed across species for specific oligo identification.

Biological researchers may obtain a list of marker genes related to human diseases or the gene expression signature derived from microarray analysis. In order to provide these researchers with useful bioinformatic tools to further investigate the genes, our SpecificDB web server provides a web interface to perform microarray data analysis and discover significant enrichment of microRNAs, transcription factors, pathways, and GOs. For example, after enrichment analysis, users can identify a panel of genes that may have significant differential expression in microarray data and have significant enrichment with a pathway. SpecificDB can bring out the specific and valid probes, siRNAs, and primers corresponding to these genes.

To demonstrate the applicability of our algorithm, *Xcc *genome analysis and genome-wide primer design have been carried out. *S. cerevisiae *and *E. coli *are usually the major contaminants in the laboratory environment, which may influence the accuracy of experiments [43,44]. To reduce the cross homology with these species, we established an integrated nucleotide sequence database consisting of four genomes (*A. thaliana*, *S. cerevisiae*, *E. coli*, and *Xcc*) for non-specific oligo filtration. Nevertheless, the results of this primer design can not be treated as completely species-specific primers. The results of PCR amplification with primers predicted by the IAB algorithm provide evidence in support of the effectiveness and accuracy of our novel algorithm.

## Conclusion

A new algorithm, the IAB algorithm, integrates ANN and BLAST to select specific oligos, and makes use of the unique markers in UMD. The IAB algorithm can effectively identify specific oligos that can serve as microarray probes, siRNAs, and primers. To demonstrate the specific oligo prediction ability of this algorithm, the whole-genome primer sets of *Xcc *strain 17 and human were designed and validated using biological PCR experiments. SpecificDB, derived from the IAB algorithm, is not only a comprehensive bioinformatic database but is also a useful web server, and is useful for functional genomics and systems biology studies.

## Methods

### The construction of UMD

The subsequences, with lengths between 10 and 26 nucleotides, of gene sequences in the database were identified by encoding. Figure 1a shows that a subsequence slides the window (one nucleotide at a time) along the TC sequence and a stack of subsequences is then collected. Every subsequence is encoded using the following formula:

code=∑i=1lci×4i−1
 MathType@MTEF@5@5@+=feaafiart1ev1aaatCvAUfKttLearuWrP9MDH5MBPbIqV92AaeXatLxBI9gBaebbnrfifHhDYfgasaacH8akY=wiFfYdH8Gipec8Eeeu0xXdbba9frFj0=OqFfea0dXdd9vqai=hGuQ8kuc9pgc9s8qqaq=dirpe0xb9q8qiLsFr0=vr0=vr0dc8meaabaqaciaacaGaaeqabaqabeGadaaakeaacqWGJbWycqWGVbWBcqWGKbazcqWGLbqzcqGH9aqpdaaeWbqaaiabdogaJnaaBaaaleaacqWGPbqAaeqaaOGaey41aqRaeGinaqZaaWbaaSqabeaacqWGPbqAcqGHsislcqaIXaqmaaaabaGaemyAaKMaeyypa0JaeGymaedabaGaemiBaWganiabggHiLdaaaa@4347@

Where c_*i *_is 0, 1, 2, or 3 for A, C, G or T at the *i*-th base of the subsequence and *l *is the length of the subsequence. For example, a sequence such as ACGTC has the coding number of 0×4^0 ^+ 1×4^1 ^+ 2×4^2 ^+ 3×4^3 ^+ 1×4^4 ^= 484 and *l *= 5. Using this encoding formula, subsequences of different DNA sequences have different coding numbers.

We used the hash table algorithm to obtain the positions of all unique subsequences that were stored in UMD. A flowchart depicting the procedures is shown in Figure 2, which indicates that the subsequences for all of the genes in the database were encoded and placed in the hash table using the coding number of the subsequences as the hash key. Moreover, if the subsequence appears only once (frequency is one), then the subsequence is unique with at least one nucleotide mismatch to all of the other sequences in the entire sequence database.

We created 10-mer ~ 26-mer marker tables and stored the locations of all unique subsequences with 10-mer ~ 26-mer for every THC sequence. The element of ANN's input vector is the density of unique subsequences (*U*_*d*_) of an oligo. The parameter *U*_*d *_is defined as follows:

Ud=NuniqueL
 MathType@MTEF@5@5@+=feaafiart1ev1aaatCvAUfKttLearuWrP9MDH5MBPbIqV92AaeXatLxBI9gBaebbnrfifHhDYfgasaacH8akY=wiFfYdH8Gipec8Eeeu0xXdbba9frFj0=OqFfea0dXdd9vqai=hGuQ8kuc9pgc9s8qqaq=dirpe0xb9q8qiLsFr0=vr0=vr0dc8meaabaqaciaacaGaaeqabaqabeGadaaakeaacqWGvbqvdaWgaaWcbaGaemizaqgabeaakiabg2da9maalaaabaGaemOta40aaSbaaSqaaiabdwha1jabd6gaUjabdMgaPjabdghaXjabdwha1jabdwgaLbqabaaakeaacqWGmbataaaaaa@3B5C@

where *N*_*unique *_is the number of unique subsequence in the oligo (Figure 1a) and *L *is the length of the oligo. According to the previous report [1], the larger value of *U*_*d *_indicates sequence specificity. Therefore, we used *U*_*d *_as the element of ANN's input vector.

### Calculation of input vector for ANN from UMD

The input vector X is defined as follows:

X= 〈Ud(10−mer),Ud(11−mer), ...,Ud(26−mer)〉
 MathType@MTEF@5@5@+=feaafiart1ev1aaatCvAUfKttLearuWrP9MDH5MBPbIqV92AaeXatLxBI9gBaebbnrfifHhDYfgasaacH8akY=wiFfYdH8Gipec8Eeeu0xXdbba9frFj0=OqFfea0dXdd9vqai=hGuQ8kuc9pgc9s8qqaq=dirpe0xb9q8qiLsFr0=vr0=vr0dc8meaabaqaciaacaGaaeqabaqabeGadaaakeaacqqGybawcqGH9aqpcqqGGaaidaaadaqaaiabdwfavnaaDaaaleaacqWGKbazaeaacqGGOaakcqaIXaqmcqaIWaamcqGHsislcqWGTbqBcqWGLbqzcqWGYbGCcqGGPaqkaaGccqGGSaalcqWGvbqvdaqhaaWcbaGaemizaqgabaGaeiikaGIaeGymaeJaeGymaeJaeyOeI0IaemyBa0MaemyzauMaemOCaiNaeiykaKcaaOGaeiilaWIaaGjbVlabc6caUiabc6caUiabc6caUiabcYcaSiabdwfavnaaDaaaleaacqWGKbazaeaacqGGOaakcqaIYaGmcqaI2aGncqGHsislcqWGTbqBcqWGLbqzcqWGYbGCcqGGPaqkaaaakiaawMYicaGLQmcaaaa@5A7A@

where  Ud(N−mer)
 MathType@MTEF@5@5@+=feaafiart1ev1aaatCvAUfKttLearuWrP9MDH5MBPbIqV92AaeXatLxBI9gBaebbnrfifHhDYfgasaacH8akY=wiFfYdH8Gipec8Eeeu0xXdbba9frFj0=OqFfea0dXdd9vqai=hGuQ8kuc9pgc9s8qqaq=dirpe0xb9q8qiLsFr0=vr0=vr0dc8meaabaqaciaacaGaaeqabaqabeGadaaakeaacqqGGaaicqWGvbqvdaqhaaWcbaGaemizaqgabaGaeiikaGIaeeOta4KaeyOeI0IaemyBa0MaemyzauMaemOCaiNaeiykaKcaaaaa@3809@ is the *U*_*d *_of N-mer subsequence in an oligo. In Figure 1a, the solid triangles mark the starting position of the unique subsequences in an N-mer oligo, and the count of the solid triangles is the number of unique subsequences. We then used the pre-established UMD to identify the location of a unique subsequence (solid triangle) and calculate *U*_*d *_immediately without searching the entire HGI or RGI database.

### Construction of data sets for training ANN

We applied the previous calculation of input vector to create the training set from the HGI database, and a k-fold validation method was employed to improve the prediction performance [29]. Therefore, 10 original data sets were created according to the prefix, including the first 6 characters of the THC ID number, such as THC180~THC189.

All of the THC sequences with the same prefix were sorted out and put together as a data set. Then, we randomly selected 200 THC sequences from each original data set to create 10 data source (DS) sets, including DS_THC180_, DS_THC181_,..., DS_THC189_. Each data source set contained 200 THC sequences with the same prefix. For example, the data source set DS_THC181 _contained 200 THC sequences randomly selected from THC1810000 to THC1819999. In this study, without losing generality, we chose DS_THC180 _as the test data source and the remainder {DS_THC181_, DS_THC182_,..., DS_THC189_} as the training data source.

#### Training set

To construct the training set (TS), 100 70-mer oligos were randomly selected from each THC sequence from the training data source. Then, a total of nine training sets were derived from the corresponding DS, which were TS_THC181 _(from DS_THC181_), TS_THC182 _(from DS_THC182_)... and TS_THC189_(from DS_THC189_). Since every DS had 200 THC sequences, it created a large training set containing 20,000 70-mer oligos. The training execution time and prediction performance were considered. We then randomly selected 50 THC sequences from DS to produce 5,000 oligos in every TS.

#### Test set

To construct the test set, 100 70-mer oligos were randomly selected from each THC sequence of test data source DS_THC180_. Thus, the test set was an assembly of 20,000 oligos, because there were 200 THC sequences in DS_THC180_.

#### Validation set

In order to understand whether the prediction has generality on large-scale databases, we randomly selected two 70-mer oligos from every THC sequence in the entire HGI database, but skipped oligos with any base ambiguity symbol, to construct the validation set. The validation set had 389,146 oligos and covered 93.6% of the HGI database. The training set described above was subjected to various conditions such as number of hidden layer nodes, learning rate, and momentum, to obtain trained ANNs. The trained ANNs were further applied to both the test and validation sets and were checked to see if the results had the same trend. If the results of test and validation sets had inconsistent trends, these results were filtered out. Finally, we selected the ANN with the best performance from the trained ANNs with consistent trends for both the test and validation sets.

#### ANN training

The Java Object Oriented Neural Engine [45], an open source project that provides a highly adaptable ANN for Java programmers, was included in our programs. The training function we used was a batch-mode training algorithm and the training procedure was terminated when the number of iterations exceeded the maximum training epoch.

We employed the sigmoid activation function as both an input and output layer. The sigmoid function's output was smoothly limited within the range of 0 to 1. The hidden layer was the logarithmic layer, which prevented the saturation of the processing elements of a layer under a lot of connected input synapses, or under input values very close to the limits 0 and 1. The momentum was set to 0.5 and the learning rate was set to 0.1 in this study.

An ANN with one hidden layer was selected and the number of hidden layer nodes was determined based on the classification performance on training data. We trained four ANNs with different numbers of hidden layer nodes (4, 10, 16, and 22 nodes), and then selected the best ANN using RMSE [28]. The RMSE was defined as follows:

RMSE = ∑i=1n(yi−y^i)2n
 MathType@MTEF@5@5@+=feaafiart1ev1aaatCvAUfKttLearuWrP9MDH5MBPbIqV92AaeXatLxBI9gBaebbnrfifHhDYfgasaacH8akY=wiFfYdH8Gipec8Eeeu0xXdbba9frFj0=OqFfea0dXdd9vqai=hGuQ8kuc9pgc9s8qqaq=dirpe0xb9q8qiLsFr0=vr0=vr0dc8meaabaqaciaacaGaaeqabaqabeGadaaakeaacqqGsbGucqqGnbqtcqqGtbWucqqGfbqrcqqGGaaicqGH9aqpcqqGGaaidaGcaaqaamaalaaabaWaaabCaeaacqqGOaakieGacqWF5bqEdaWgaaWcbaGaemyAaKgabeaakiabgkHiTiqbdMha5zaajaWaaSbaaSqaaiabdMgaPbqabaGccqGGPaqkdaahaaWcbeqaaiabikdaYaaaaeaacqqGPbqAcqGH9aqpcqqGXaqmaeaacqqGUbGBa0GaeyyeIuoaaOqaaiabd6gaUbaaaSqabaaaaa@4632@

where n is the number of input vectors, *y*_*i *_is the output value of every input vector, and y^i
 MathType@MTEF@5@5@+=feaafiart1ev1aaatCvAUfKttLearuWrP9MDH5MBPbIqV92AaeXatLxBI9gBaebbnrfifHhDYfgasaacH8akY=wiFfYdH8Gipec8Eeeu0xXdbba9frFj0=OqFfea0dXdd9vqai=hGuQ8kuc9pgc9s8qqaq=dirpe0xb9q8qiLsFr0=vr0=vr0dc8meaabaqaciaacaGaaeqabaqabeGadaaakeaacuWG5bqEgaqcamaaBaaaleaacqWGPbqAaeqaaaaa@2FBE@ is the desired output (from BLAST) of every input vector.

#### Integration of ANN and BLAST (IAB algorithm)

In this study, we designed an algorithm integrating ANN and BLAST (IAB algorithm) to identify specific N-mer oligos with high efficiency. The pseudo code of the IAB algorithm is shown in Table 4 and the architecture of IAB is shown in Figure 1. The cross homology of a specific oligo was determined by the similarity between the specific oligo and its best homology in the non-target sequences, and it was calculated by BLAST. The ANN score was the output value of the trained ANN and could indicate the cross homology.

**Table 4:** The integration of ANN and BLAST (IAB algorithm).

**IAB**^a ^(*T*,*N*,*F*)^b^	
1.	*size *← length [*T *] - *N*
2.	Oligo *allOligo *[*size*]/* the data structure Oligo includes score and sequence*/
3.	***for ****pos *← 1 ***to ****size*
4.	***do ****allOligo *[*pos*].*sequence *← *T*.substring (*pos, pos + N*)
5.	/* calculate the ANN score for each sliding oligo using the trained ANN */
6.	*allOligo *[*pos*]*.score *= CalScoreByNN ^c ^(*allOligo *[*pos *])
7.	sort the *allOligo *array into non-decreasing order by ANN score
8.	*lowestSim *← 1.0
9.	***for ****p *← 0 ***to ***(*size *• *F*^d^)
10.	***do ****oligo *← *allOligo *[*p*].*sequence*
11.	*similarity *← CalSimByBlast ^e ^(*oligo*)
12.	***if ****similarity < lowestSim*
13.	***then ****bestOligo *← *oligo*
14.	*lowestSim *← *similarity*
15.	***if ****lowestSim *<*TH*_*sim*_^f^
16.	***then return ****bestOligo*
17.	***return ****bestOligo*

^a^IAB, integration of artificial neural network (ANN) and basic local alignment search tool (BLAST). ^b^The input parameters are as follows: the THC sequence *T*, the oligo length *N*, and the sensitivity factor *F*; ^c^CalScoreByNN procedure calculates the ANN score (indicates the cross homology) by trained ANN; ^d^The sensitivity factor is set as 0.2, 0.3, and 0.4; ^e^CalSimByBlast procedure calculates the cross homology by WU-BLAST; ^f^The cross homology threshold (*TH*_*sim*_) used for 70-mer and 50-mer was 0.7 and that used for 25-mer was 0.8.

A brief description of the IAB algorithm is as follows: (1) take one THC sequence as input and calculate the ANN score for each sliding N-mer oligo of the input using the trained ANN (Lines 1 ~ 5); (2) calculate the cross homology by WU-BLAST (oligo with the lowest ANN score is evaluated first) (Lines 6 ~ 15); and (3) the procedure will be finished when the first specific oligo is found; otherwise, a certain percentage (sensitivity factor) of the oligos will be screened.

The sensitivity factor was defined as the maximum percentage of sliding oligos in the input gene sequence that would be screened by BLAST. In this study, we randomly selected 100 THC sequences on which to perform our algorithm. The cross homology threshold used for the 70-mer and 50-mer was 70% while that for 25-mer was 80%.

To investigate the performance with and without ANN under the same conditions (e.g. the length of oligos, test set, and genome), we carried out BLAST search and compared it with the results derived from the IAB algorithm. The procedures for pure BLAST search are as follows: (1) for each sliding N-mer oligo of the input THC sequence, the cross homology is calculated by WU-BLAST; (2) if the cross homology of any oligo is less than the threshold (*i.e*. the specific oligo), the first specific oligo is found and the procedure is finished; (3) if the procedure cannot find any specific oligo, it will screen all sliding N-mer oligos and then return the oligo with the lowest cross homology. Pure BLAST is similar to the IAB algorithm (Table 4) but skips the calculation of the ANN scores. Although calculation of the ANN scores requires extra execution time, it could save more execution time by decreasing the number of BLAST calculations needed because the ANN score can help filter out non-specific oligos. Furthermore, in order to understand the performance of BLAST search with *U*_*d*_, we have implemented a program that can sort the oligo candidates based on the summation of 10-mer ~ 26-mer *U*_*d *_and BLAST each oligo candidate on the sorted list until a suitably specific oligo has been found.

### Probe design procedure

The rules described by Chang and Peck [1] for probe selection were adopted in this study. Under the selection rules, sequence sections were discarded if they met any of the following criteria: (a) number of any single bases (As, Cs, Ts or Gs) exceeded half of the section length; (b) the length of any contiguous As, Cs, Ts, or Gs exceeded a quarter of the section length; (c) GC content was under 40% or over 60%; or (d) no self-complementary region within the sequence section. The sequence sections that do not meet the above criteria are considered as candidate probes for further selection by our IAB algorithm. The ANN predicted the 10 most specific oligos for each THC, for which we then used WU-BLAST to calculate the cross homology. Finally, we filtered the oligos having high cross homology and displayed the top four probes.

### siRNA design procedure

We used the UMD to get 19-mer unique markers for each THC sequence. Suppose that there were N unique oligos of 19-mer in a THC sequence, the ANN scores of these N oligos were calculated, and the N/3 oligos with the lowest ANN scores were selected because ANN scores indicate cross homology. Then we used the eight criteria described by Reynolds *et al*. to compute siRNA score [35]. We selected the top two oligos by the siRNA scores to calculate cross homology by WU-BLAST. Finally, we chose the best oligo using the cross homology of each oligo.

### Primer design procedure

The primer design procedure was as follows: (a) used primer3 program [46] to produce primer candidates; (b) calculated the ANN score for all candidates; (c) selected top 10 primers with the highest ANN score to calculate the cross homology by WU-BLAST; and (d) the primer with the lowest cross homology was selected.

### The polymerase chain reaction for Xcc primer sets

The PCR amplifications were carried out in a 50 μl reaction mixture containing 1X buffer, 200 μM dNTP, 0.2 μM of each primer, 2 μl DMSO, 200 ng genomic DNA templates, and 2 units of thermostable polymerase (proTag plus; Protech Technology Enterprise Co., Taiwan). The PCR conditions were as follows: the 1st cycle, 94°C for 5 min, 60°C for 1 min then 72°C for 1 min; the 2^nd ^- 30^th ^cycle: 94°C for 1 min, 60°C for 1 min then 72°C for 1 min, and a final extension for 10 min at 72°C. The PCR products were then subjected to 1% of agarose gel electrophoresis.

### The polymerase chain reaction for human primer sets

The PCR amplifications were carried out in a 20 μl reaction mixture containing 1X GI buffer (Takara), 200 μM dNTP, 0.2 μM of each primer, 100 ng cDNA templates of lung cancer CL1-0 cell line, and 1 units of thermostable polymerase (proTag plus; Protech Technology Enterprise Co., Taiwan). The PCR conditions were as follows: the 1st cycle, 94°C for 5 min; the 2nd – 30th cycle: 94°C for 1 min, 58°C for 1 min then 72°C for 1 min, and a final extension for 10 min at 72°C. The PCR products were then subjected to 2% of agarose gel electrophoresis.

## Abbreviations

ANN: artificial neural network; BLAST: basic local alignment search tool; DS: data source; GO: gene ontology; HGI: human gene index; IAB: integration of ANN and BLAST; oligo: oligonucleotide; PCR: polymerase chain reaction; RGI: rat gene index; RMSE: root mean square error; siRNA: small interfering RNA; SpecificDB: specific oligo web server; TC: tentative consensus; THC: tentative human consensus; TIGR: the Institute for Genomic Research; TS: training set; *U*_*d *_: the density of unique subsequences; UMD: unique marker database; *Xcc: Xanthomonas campestris pv. Campestris*.

## Authors' contributions

CCL, PCC, and JJWC conceived and designed the methods. CCL wrote the software program. CCL, KCL, PCY, and JJWC wrote the paper. CCL and WSEC conceived and designed the computational analysis. CCL, JCC and MTY designed and performed the biological experiments.

## Supplementary Material

Additional file 1 Excel spreadsheet, Supplemental Table S1. The genome-wide primer sets for the *Xcc *strain 17. Click here for file
